# Rapid Recent Warming of Coral Reefs in the Florida Keys

**DOI:** 10.1038/srep16762

**Published:** 2015-11-16

**Authors:** Derek P. Manzello

**Affiliations:** 1 Atlantic Oceanographic and Meteorological Laboratories (AOML), NOAA, 4301 Rickenbacker Cswy., Miami, FL 33149.

## Abstract

Coral reef decline in the Florida Keys has been well-publicized, controversial, and polarizing owing to debate over the causative agent being climate change versus overfishing. The recurrence of mass bleaching in 2014, the sixth event since 1987, prompted a reanalysis of temperature data. The summer and winter of 2014 were the warmest on record. The oldest known *in-situ* temperature record of any coral reef is from Hens and Chickens Reef (H&C) in the Florida Keys, which showed significant warming from 1975–2014. The average number of days ≥31.5 and 32^o^C per year increased 2670% and 2560%, respectively, from the mid-1990 s to present relative to the previous 20 years. In every year after 1992 and 1994, maximum daily average temperatures exceeded 30.5 and 31°C, respectively. From 1975–1994, temperatures were <31 °C in 61% of years, and in 44% of the years prior to 1992 temperatures were <30.5 °C. The measured rate of warming predicts the start of annual bleaching between 2020 and 2034, sooner than expected from climate models and satellite-based sea temperatures. These data show that thermal stress is increasing and occurring on a near-annual basis on Florida Keys reefs due to ocean warming from climate change.

The degradation of coral reefs throughout the Caribbean since the 1980 s has been alarming^1^,^2^,^3^,^4^,^5^,^6^. Some workers have attributed this degradation and loss of coral cover to overfishing that eliminated herbivores, allowing macroalgae to proliferate, overgrow, and kill corals^7^. Others have argued that the increase in coral disease and warm water coral bleaching as a result of climate change are the causative agents of this decline and the increases in macroalgae are an effect rather than a cause of coral mortality^8^. Warm water coral bleaching events have increased in frequency and severity over this time period and have inarguably been a major factor in this decline^9^,^10^,^11^,^12^. Bleaching from a warming ocean is now considered one of the biggest threats to the continued existence of coral reefs over the coming decades as the rate of ocean warming predicts that annual mass bleaching will occur on every reef across the globe somewhere around the year 2050^13^. The coral reefs of the Florida Keys have reflected the global trend of an increase in mass coral bleaching. Beginning in 1987, there have been six mass coral bleaching events that impacted the entire Florida Keys^14^,^15^. The most recent event took place in the summer of 2014 (Fig. 1). From 2006–2013, there were qualitative observations of ‘moderate’ warm water bleaching every year except one^16^. The only year when reports of bleaching were low was the only year over this period that was notably cooler than the climatology at Molasses Reef (MLRF) in 2013 (ref. 17).

There is a paucity of long-term, *in-situ* temperature data for coral reefs. The most continuous record in the Florida Keys was initiated in 1988 after the first mass bleaching event had taken place in 1987. This record is from the shallow lighthouse (~5 m) on MLRF, which is an offshore reef in the upper Florida Keys (Fig. 2). The oldest record is from Hens and Chickens Reef (H&C), an inshore patch reef, and was collected by J. Harold Hudson and colleagues from 1975–2007 (ref. 18). This is the oldest known temperature record collected on any coral reef worldwide. Time-series temperature measurements do exist farther back in time, but these were collected from adjacent, non-reef areas^19^. Other *in-situ* records were established in the late 1980 s and early 1990 s on existing structures at offshore reef sites SE of Miami to Key West, as well as the Dry Tortugas^15^. These records, unfortunately, have large gaps, and two of these structures were damaged or destroyed by hurricanes in 2004 and 2005, ending data collection.

The recurrence of mass coral bleaching in 2014 prompted a reanalysis of the long-term *in-situ* temperature data that was initially studied from 1988–2005 for MLRF^15^. These data are supplemented with additional analyses of the H&C dataset with inclusion of new data from 2014 to determine how the warming shown by Kuffner *et al.*^18^ corresponded to an increase in the number of days ≥30.5, 31, 31.5, and 32 °C each year. We focused on August and September because this is when data were most complete, as well as when temperatures reach seasonal maxima^15^. These temperatures were chosen because number of days ≥30.5 °C correlated with mass bleaching events in 1997, 1998, and 2005, one day ≥31 °C was predicted to cause bleaching at MLRF^15^, and Jaap^20^ observed bleaching when temperatures reached 32 °C. Lastly, these long-term historical records are considered alongside *in-situ* temperatures from paired inshore and offshore sites in the upper, middle, and lower Florida Keys from 2010–2012 (Fig. 2).

## Results and Discussion

The summer of 2014 and winter of 2013–2014 were the warmest on record for MLRF (Fig. 3). Prior to 2014, the warmest winter and summer on record were in 1997 at the start of the extreme 1997–1998 El Niño warming event. H&C exhibited a significant warming trend from 1975–2014 for number of days ≥30.5, 31, 31.5, and 32 °C, illustrating that the exposure of corals to thermally stressful conditions has increased significantly over the past 40 years (Fig. 4, Table S1). These trends held when the anomalous El Niño years of 1997–1998 were removed, except for days ≥32 °C. The lack of significance for days ≥32 °C is likely because only three out of the 26 years of data had non-zero values (1980, 2005, 2014). Seventeen of the past 27 years at MLRF were warmer than the climatology. In spite of this, there were no trends in: 1) the entire MRLF dataset (all data with time), 2) monthly mean temperatures (each month by year), or 3) days ≥30, 30.5, and 31 °C. However, when the El Niño years of 1997–1998 were removed, the trends for days ≥30.5 and 31 °C with time were positive and significant (Table S1). As previously mentioned, the MLRF dataset was initiated after the first mass bleaching event in the Florida Keys and temperatures at H&C warmed considerably in the early 1990 s (Fig. 4, Table 1). This coupled with the anomalously warm El Niño years of 1997–1998 could explain the muted warming signal for MLRF, as it likely biased the first 10 years of data towards higher values.

In every year of available data since 1996, maximum daily temperatures at H&C were ≥31 °C (Fig. 4, Table 1). Prior to 1995, maximum daily sea temperatures were <31 °C in 11 of the 18 years of available data (61%) from 1975–1994. In almost half of the years from 1975–1992 (44%, 7 of 16 yrs), maximum temperatures were <30.5 °C, but then exceeded this value every year beginning in 1993. Temperatures ≥32 °C were recorded for only one day in total from 1975–1996 (0.05 days year^−1^), but increased 2560% to a frequency of 1.33 days year^−1^ thereafter (Table 1). The average number of days ≥31.5 °C per year increased 2670% over 1993–2014 when compared to 1975–1992.

The differences in number of hours >31 and 32 °C between our study sites over three years (2010–2012) and values at H&C and other reef sites from 1977–1980 were dramatic (Table 2). Note that hourly average temperatures for H&C are only available from 1977–1980 as reported in Hudson^21^. The offshore sites in 1977–1980 never experienced temperatures >31 °C, whereas the three offshore sites in 2010–2012 experienced 84–664 hours >31 °C per year and routinely experienced temperatures >32 °C. In 2010, the offshore sites in the upper and middle Keys experienced 60 and 105 hours >32 °C, respectively. The cumulative number of hours >32 °C over three years at the three inshore sites was 850–889 hours, whereas there were 904 hours over four years in 1977–1980 at Snake Creek channel. The inshore sites in this study experienced a comparable dosage of cumulative thermal stress over three years (2010–2012) as Snake Creek did over four years in the late 1970 s. Snake Creek was specifically chosen by Hudson^21^ to illustrate its thermally unsuitable nature for corals and the coral transplanted there bleached from warm water stress. This site gets hot because it is shallow and subject to the outflow of Florida Bay waters, which heat up considerably during summer^21^,^22^.

Table 3 shows the year when the duration of temperatures that were associated with major bleaching events in 1997, 1998, 2005, and 2014 are predicted to occur annually based on the regressions of Fig. 4 and Table S1. The time-temperature bleaching curve presented in Manzello *et al.*^15^ for MLRF predicts that bleaching will occur when daily average temperatures are ≥30.5 °C for 16 days, or 1 day ≥31 °C. A minimum of 23 days ≥31 °C were associated with major bleaching at H&C in 1997, 1998, 2005, and 2014. When just the H&C data were used, bleaching is expected to occur annually within the next 20 years at this site (2021–2034). The most conservative estimate for H&C, using the values for the missing years that were estimated from nearby Snake Creek (see methods), predicts annual bleaching in 2045. When the values from the upper keys inshore site that is 1.4 km away from H&C were included to fill in missing years from 2010–2013, the regression with time changed little, predicting annual bleaching from 2027–2035. Annual bleaching was predicted to occur for MLRF no later than 2020.

Annual bleaching is predicted to occur later than 2040 based on satellite sea surface temperatures (SST) and the most severe emission scenario (RCP 8.5) for reefs in the Florida Keys near H&C and MLRF^23^. All the predictions based on *in-situ* temperatures are much earlier than this, except for the most conservative estimate of 2045. This suggests that the in-water thermal environment in the Florida Keys may be more complex and warmer than what is captured from satellite-based SSTs. The presence of an inverse thermocline due to dense, salty, and hot waters exiting Florida Bay is one such mechanism whereby sea temperatures are higher near the corals than the surface and may be a factor in these differences^24^.

During the first widespread bleaching event in 1987, temperatures at Alligator Reef, an offshore reef site SW of H&C, were 30 °C or slightly greater for about a month^25^. These temperatures were measured at MLRF in 18 of 27 years from 1988–2014. The value that co-occurred with the 1990 mass bleaching (max. running 30-day mean = 30.2 °C)^14^ occurred in 14 of the 27 years and 8 of the past 11 years (Fig. 3). These observations suggest that the bleaching thresholds for MLRF could be conservative and that stressful conditions may manifest as low as monthly temperatures of 30 °C. Heat stress may be more chronic than what is currently appreciated and there could be a shifting baseline whereby summertime bleaching has become the norm^26^. It’s likely no coincidence that 2013 was the first year since 1996 that was considerably cooler than the climatology during summer (Fig. 3, max temperature = 29.5 °C) and is the only year in recent record when reports of bleaching were low^16^. The highest growth and calcification rates in *Orbicella faveolata* from 2004–2013 and *Porites astreoides* from 2001–2013 occurred during this cool period^17^,^27^.

Bleaching thresholds may increase as field and experimental evidence have shown that repeated warm water stress or routine exposure to high and variable temperatures can lead to elevated thermal tolerance due to adaptation and acclimatization^28^,^29^,^30^,^31^. As summarized in Chollett *et al.*^32^, the rate of seasonal warming is also important such that a rapid rate of temperature increase leads to a decreased potential for the holobiont to cope with thermal stress, aggravating the bleaching response and post-stress fate of the reef. The rate of seasonal warming at MLRF was high during all bleaching years (Fig. S1), which may have deterred any uptick in the bleaching threshold to-date. The potential for adaptation and acclimatization will likely not be able to compensate for unabated warming^31^. Furthermore, thermal stress does not just elicit coral bleaching and mortality, but also results in increases in coral disease, while causing declines in reproductive output, linear extension, and coral calcification^17^,^27^,^33^,^34^.

The interaction of heat stress and irradiance ultimately drives the bleaching response^35^, but all large-scale coral bleaching episodes over the past 30 years have been associated with warmer than average temperatures^11^. Local-scale variability during bleaching can be due to differential irradiance, as well as differential susceptibilities of coral taxa to heat stress^36^,^37^. In the Caribbean, however, the most important reef-building taxa, namely the acroporids and *Orbicella annularis* spp. complex, have experienced the greatest declines owing mainly to bleaching and disease^8^,^38^. The weedy taxa, such as *Porites astreoides*, which have increased in relative abundance with the losses of the aforementioned species, have little potential to construct or maintain architecturally complex framework structures that are vital to ecosystem function^39^. The flattening of reefs, or loss in architectural complexity, has already occurred across many Caribbean reefs and many reefs are presently in a state of accretionary stasis, or net erosional, including those in the Florida Keys^6^,^40^,^41^. Thus, the differential susceptibilities to warm-water bleaching and persistence of certain taxa has had little impact in slowing the large scale degradation of Caribbean coral reefs.

The shallow back-reef pools in Ofu, American Samoa have been of recent interest because >50 coral species are able to tolerate short periods of high temperatures in this location^42^. There were 225 total days when temperatures were >32 °C from 2000–2007 (ref. 43). These temperatures, however, only occurred on average 2–3 hours per day. If we use the maximum value of 3 hours per day, this yields 84 hours per year >32 °C. Temperatures were >32 °C for 35 days during the summer of 1998–1999 and the mean duration of these warm water periods was 2.4 hours^43^. This too yields 84 hours >32 °C. The Florida Keys sites had similar maximum temperatures in 2010–2012, but the mean summer temperatures in Florida were generally >1 °C warmer than Samoa (Table 4). At the inshore sites, the number of hours >32 °C were much greater than Samoa, as the range was 133–404 hours per year at the reef sites from 2010–2012 (Table 2). Tennessee Reef, an offshore reef site where temperatures are more buffered than inshore from extremes, experienced 105 hours >32 °C in 2010. The Florida Keys routinely experienced acute temperature stress similar to Ofu, but also greater chronic heat stress given the higher mean temperatures both inshore and offshore.

The acceleration of both acute and chronic heat stress over the past ~30 years coincides with the degradation and loss of coral cover in the Florida Keys, suggesting that climate change and ocean warming are playing a crucial role in this degradation. These data suggest that the iconic coral reefs of the Florida Keys are routinely experiencing high levels of thermal stress on a near-annual basis. Given the magnitude of the recent *in-situ* thermal stress relative to that from the late 1970 s, the impact of heat stress in the Florida Keys may be underappreciated (Table 2). Chronic warm-water stress could help explain the continued decline of the *Oribicella annularis* species complex since the 1997–98 El Niño, the very low coral cover at most reefs other than the inshore patch reefs, and little recovery^44^. The synchronous decline of coral populations both within and outside of protected areas^45^ coupled with the data presented here provide further evidence that the loss of coral cover in the Florida Keys is a result of ocean warming and climate change.

## Materials and Methods

**Figure 2:**
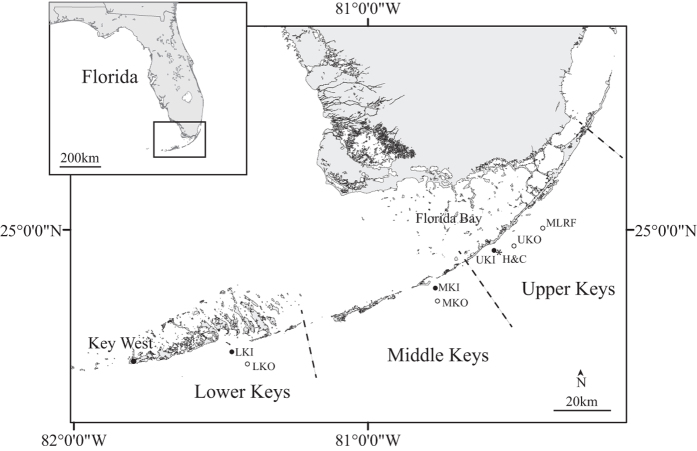
Sites in Florida Keys where temperature measurements were obtained. Open circles are offshore sites, closed circles are inshore sites, and asterisk is Hens and Chickens. Abbreviations, MLRF, Molasses Reef (25.012°N, 80.376°W); UKO, Upper Keys Offshore (24.947^o^N, 80.502^o^W); H&C, Hens and Chickens Reef (24.933°N, 80.549°W); UKI, Upper Keys Inshore (24.939^o^N, 80.563^o^W); MKO, Middle Keys Offshore (24.767°N, 80.753°W); MKI, Middle Keys Inshore (24.812^o^N, 80.761^o^W); LKO, Lower Keys Offshore (24.551^o^N, 81.402^o^W); LKI, Lower Keys Inshore (24.597^o^N, 81.455^o^W). The map was taken from ArcGIS base layers and modified in Adobe Illustrator.

**Figure 3:**
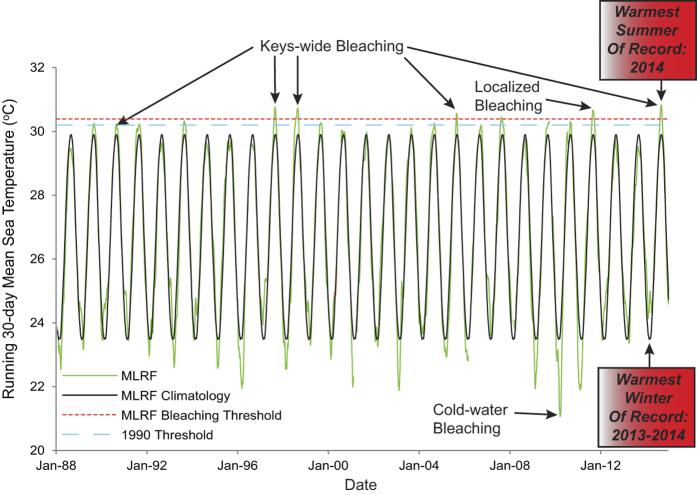
Time-series of running 30-day mean seawater temperature from Molasses Reef since 1988 plotted against climatology. Periods of known thermal stress are indicated. The MLRF threshold (red dashed line) is the coral bleaching threshold determined previously based on mass bleaching events in 1997, 1998, and 2005 (ref. 15). The 1990 threshold (blue dashed line) corresponds to temperatures that occurred when Milleporid hydrozoan corals bleached throughout the Florida Keys. Milleporid corals are known to be thermally sensitive, therefore this value may represent a threshold for these heat-sensitive corals.

**Figure 4:**
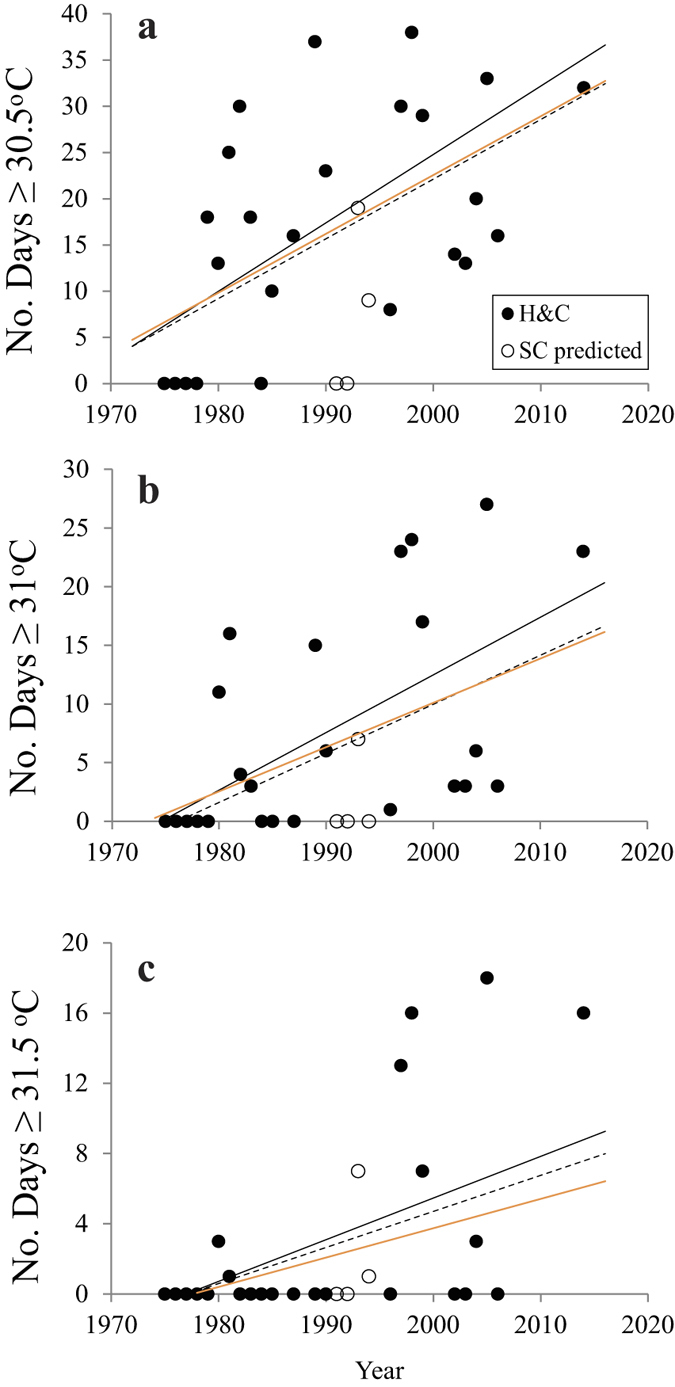
Number of days ≥30.5 (**a**), 31 (**b**), and 31.5 °C (**c**) at Hens and Chickens Reef plotted by year from 1975-2014. Solid circles are Hens and Chickens data (H&C), open circles are the points predicted by the regression with Snake Creek data (SC predicted). Poisson linear regression lines shown for H&C (solid line), H&C plus SC predicted (dashed line) and H&C with ENSO years removed (orange line).

There were three different temperature records utilized in this analysis. First, hourly sea temperatures from the MLRF Coastal Marine Automated Network (C-MAN) station were analyzed from 1988–2014 (ref. 46) (Fig. 2). The climatology and bleaching thresholds for this site were determined as previously described^15^. The bleaching thresholds were estimated as a monthly mean sea temperature ≥30.4 °C, or daily average temperatures ≥30.5 °C for 16 days, or 1 day ≥31 °C. We also calculated the rate of seasonal warming as done by Chollett *et al.*^32^ to determine how it related measured temperature stress.

Secondly, the temperature record from H&C from 1975–2006 was assessed with the inclusion of new data from 2014. Hourly temperatures are available from 1977–1980, but the remainder of the record are daily averages^18^,^21^. A temperature sensor (Seabird SBE 56) was deployed at H&C in December 2013 at the same depth of the historical thermistor (4 m) and data from August and September of 2014 are presented. Data were collected every 5 minutes and converted to hourly and daily averages. Poisson linear regression, necessary for count data^47^, was used to determine if there was a trend with time in number of days ≥30.5, 31, 31.5, and 32 °C each year. The Durbin-Watson statistic was calculated to test for temporal autocorrelation and showed that these data did not exhibit autocorrelation. These regressions were used to estimate the year when temperatures associated with prior mass bleaching events are predicted to occur annually. Statistical analyses were performed using SigmaPlot 12.

The H&C data set is incomplete for 1986, 1988, 1991–95, and 2000–2001. Also, there were gaps in 1976 and 1977. In 1976, data were unavailable from 12 September onward and in 1977 from 18 August. In 1976, temperatures had declined to <27.5 °C by 12 September, thus it was assumed they did not again increase 2.5 °C to 30 °C in what was a cool year when temperatures were seasonally declining. Furthermore, the maximum temperature in 1976 was 30.4 °C on 4 Aug and values were <30 °C from 13 Aug onward. The gap in 1977 is longer and more problematic. This gap in 1977, however, is presented by Hudson^21^ alongside temperatures from a nearby site (Peanut) that is closer to shore and experienced warmer temperatures. The site closer to shore continued to cool and maximum temperatures never reached 30 °C during the period that data were unavailable at H&C. As a result, it is highly unlikely that temperatures were >30 °C at H&C given that it was always cooler than this other site when data overlapped from 1977–1980. Thus, it was assumed temperatures were <30 °C for the gaps in data in 1976 and 1977.

We utilized data from an additional one of Hudson’s sites at Snake Creek that are available online^18^ to estimate days ≥30.5, 31, 31.5, and 32 °C at H&C from 1991–1994. Linear regression equations were used to predict H&C values from Snake Creek based on the 14 years when temperatures were collected at both sites simultaneously. The regression statistics were as follows: ≥30.5 °C, *R*^*2*^ = 0.741, *F* = 34.4, *p* < 0.001; ≥31 °C, *R*^*2*^ = 0.617, *F* = 19.3, *p* < 0.001; ≥31.5 °C, *R*^*2*^ = 0.338, *F* = 6.13, *p* < 0.05; ≥32 °C, *R*^*2*^ = 0.5, *F* = 11.96, *p* < 0.01. The regression with the Snake Creek data predicted zero days ≥32 °C from 1991–1994 years, and one and seven days ≥31.5 and 31 °C, respectively, in 1993.

Hudson^21^ quantified thermal stress as number of hours >31 and 32 °C from 1976–1980. We calculated these metrics from six paired inshore and offshore sites in the upper, middle, and lower Florida Keys from 2010–2012 (the third temperature record) for comparison (Fig. 2). These sites were carefully selected so that they had similar depths (4–6 m). Temperature was measured every 30 minutes using HOBO Pro V2 thermistors (Onset Corp.) that were ~0.5 m above the substrate attached to rebar hammered into the reef. Hens and Chickens is 1.4 km (or 0.89 miles) away from the inshore site in the upper Florida Keys (Fig. 2).

## Additional Information

**How to cite this article**: Manzello, D. P. Rapid Recent Warming of Coral Reefs in the Florida Keys. *Sci. Rep.* **5**, 16762; doi: 10.1038/srep16762 (2015).

## Supplementary Material

Supplementary Information

## Figures and Tables

**Figure 1:**
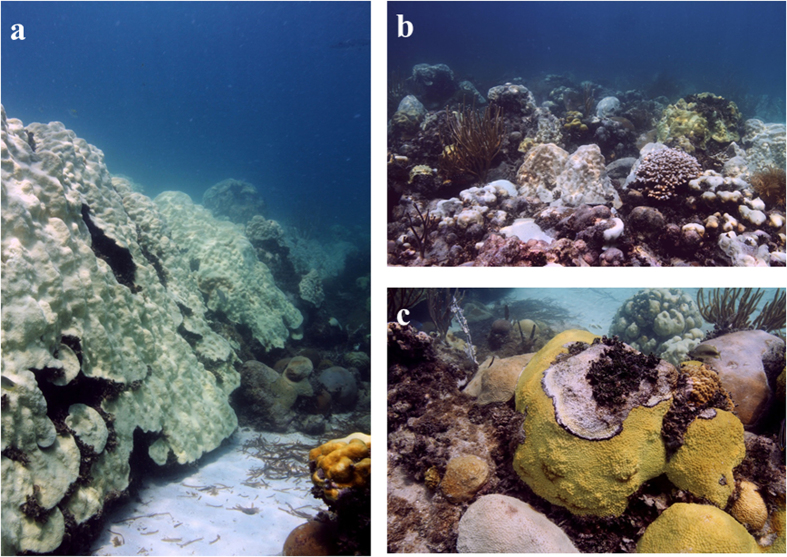
Coral bleaching in the Florida Keys during the summer of 2014. (**a**) Large colony of *Orbicella (*formerly *Montastraea*) *faveolata* completely bleached. Colony pictured is several meters in height and diameter. (**b**) Bleached reef landscape showing multiple species of affected coral. (**c**) Black band disease on colony of pale *Montastraea cavernosa*. The incidence of coral disease increases during coral bleaching events. Photographs taken by author at Cheeca Rocks, Florida Keys in September 2014.

**Table 1:**
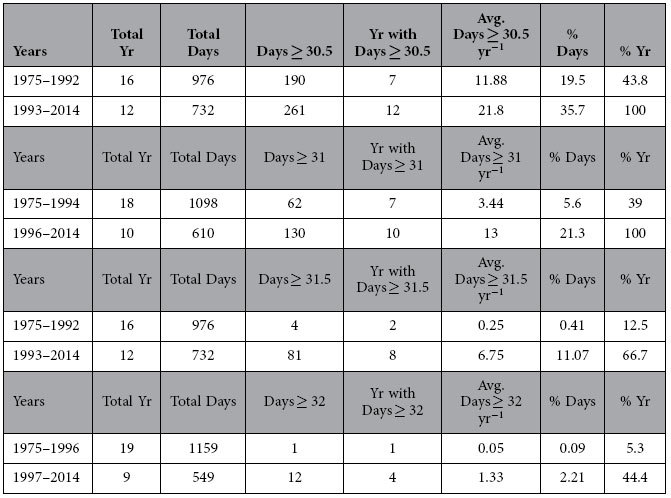
Number of years and days that daily average temperatures were ≥30.5, 31, 31.5, and 32 °C at Hens and Chickens Reef since 1975. 1992 and 1994 were last years that daily average temperatures did not exceed 30.5 and 31 °C, respectively.

**Table 2:** Comparison of thermal extremes of Hudson^21^ from 1977–1980 with recent data.

Site	Site Type	Depth (m)	Year	No. of hrs>31 °C	No. of hrs>32 °C
Snake Creek	Non-reef	2.5	1977	NR	45
(Most inshore)			1978	NR	2
			1979	NR	299
			1980	NR	558
Hens and Chickens	Coral Reef	5.7	1977	NR	0
			1978	NR	0
			1979	NR	0
			1980	NR	25
			***2014***	***805***	***104***
Midshore-Edge	Coral Reef	5.1	1977	0	0
			1978	0	0
			1979	0	0
			1980	0	0
Crocker Reef	Coral Reef	4.1	1977	0	0
(Most offshore)			1978	0	0
			1979	0	0
			1980	0	0
Upper Keys Inshore	Coral Reef	4.2	2010	989	404
(Tavernier Rocks)			2011	1124	315
			2012	605	133
Middle Keys Inshore	Non-Reef	4	2010	1050	422
			2011	1151	313
			2012	534	157
Lower Keys Inshore	Coral Reef	4.3	2010	1047	388
(Marker 50A)			2011	1224	284
			2012	790	209
Upper Keys Offshore	Coral Reef	5	2010	437	60
(Little Conch Reef)			2011	522	0
			2012	84	0
Middle Keys Offshore	Coral Reef	4.5	2010	475	105
(Tennessee Reef)			2011	390	2
			2012	ND	ND
Lower Keys Offshore	Coral Reef	4.4	2010	354	13
(Looe Key backreef)			2011	664	11
			2012	90	0

NR, data not reported. ND, no data because logger was lost. Coral reef and Non-reef sites indicated.

**Table 3:** Predicted year that temperatures will annually reach values associated with previous mass bleaching events based on Poisson Linear Regression.

Site	Threshold	ENSO Years Included	Year	ENSO Years Excluded	Year
		Equation		Equation	
H&C	23 days ≥ 31 °C	Days = 0.491*Yr – 969.1	2021	Days = 0.377*Yr – 743.9	2034
H&C + SC		Days = 0.418*Yr – 826.0	2029	Days = 0.315*Yr – 621.3	2045
H&C + SC + UKI		Days = 0.442*Yr – 873.5	2027	Days = 0.381*Yr – 752.4	2035
MLRF	16 days ≥ 30.5 °C	ns	ns	Days = 0.481*Yr – 955.5	2020
	1 day ≥ 31 °C	ns	ns	Days = 0.050*Yr – 100.1	2014

Data shown with and without strong ENSO years of 1997–1998. ns, not significant.

**Table 4:** Comparison of summertime mean, standard deviation and maximum temperatures in Florida Keys and American Samoa.

Site	Site Type	Depth	Year	Mean (SD)	Max	Max 30-d
Upper Keys Inshore	Coral Reef	4.2	2010	30.81 (1.075)	32.85	31.69
(Tavernier Rocks)			2011	30.97 (0.955)	33.94	31.79
			2012	30.19 (1.236)	33.24	31.29
Middle Keys Inshore	Non-Reef	4	2010	30.83 (1.084)	34.05	31.66
			2011	30.97 (0.992)	33.52	31.73
			2012	30.19 (1.242)	33.73	31.35
Lower Keys Inshore	Coral Reef	4.3	2010	30.83 (0.909)	33.18	31.58
(Marker 50A)			2011	30.99 (0.874)	32.77	31.74
			2012	30.31 (1.354)	33.50	31.53
Upper Keys Offshore	Coral Reef	5	2010	30.33 (0.720)	32.85	30.90
(Little Conch Reef)			2011	30.45 (0.696)	31.94	30.91
			2012	29.63 (0.867)	31.59	30.41
Middle Keys Offshore	Coral Reef	4.5	2010	30.42 (0.749)	33.13	31.01
(Tennessee Reef)			2011	30.37 (0.707)	32.23	30.98
			2012	ND	ND	ND
Lower Keys Offshore	Coral Reef	4.4	2010	30.26 (0.596)	32.12	30.70
(Looe Key backreef)			2011	30.51 (0.729)	32.25	31.02
			2012	29.48 (0.810)	31.92	30.15
Ofu, American Samoa	Pool A	1.1	1998/99	29.3 (0.88)	34.5	NR
			1999/00	29.1 (0.92)	33.7	NR
Pool B	1.9	1999/00	29.3 (0.55)	31.9	NR	

Data collected every 30 min in Florida and American Samoa^41^. Temperatures are degrees Celsisus, depth is in meters. SD, standard error, Max 30-d is maximum 30-day running mean daily average temperature. ND, no data. NR, not reported.
