# Supplementary material for: Rapid Recent Warming of Coral Reefs in the Florida Keys
Source: Sci Rep. 2015 Nov 16;5:16762. doi: 10.1038/srep16762 (PMC4645222; doi:10.1038/srep16762)
Supplement: Supplementary Information [file srep16762-s1.doc]

**Rapid recent warming of coral reefs in the Florida Keys**

Derek P. Manzello

**Supplementary Information**

***Supplemental Figure***

**Supplementary Figure 1 |** **Rate of seasonal temperature increase plotted against acute thermal stress.** Values calculated according to Chollett et al. (2014). Closed circles are non-bleaching years, open circles are bleaching years with year denoted

***Supplemental Table***

**Supplementary Table 1 |** **Results of Poisson Linear Regression for number of days ≥ 30.5, 31, 31.5, and 32^o^C vs. time in years.** Data presented for just Hens and Chickens (H&C, N = 24 years from 1975-2014), Hens and Chickens with missing years 1991-94 filled in by linear regression with data from Snake Creek (H&C + SC, N = 28 years from 1975-2014), Hens and Chickens with Snake Creek and additional data collected from UKI in 2010-2013 (H&C + SC + UKI, N = 32 years from 1975-2014), and Molasses Reef (MLRF, N = 22 years from 1988-2014). Regression data shown with and without strong ENSO years of 1997-1998. ns, not significant. There were zero days ≥ 31.5 ^o^C for MLRF over entire record.

**ENSO Years Included ENSO Years Excluded**

**Data source # days *F* *R*^2^ Slope *p*-value *F* *R*^2^ Slope *p*-value**

H&C ≥ 30.5 11.7 0.347 0.740 < 0.01 8.0 0.287 0.637 < 0.05

≥ 31 16.3 0.425 0.491 < 0.001 7.9 0.282 0.377 < 0.05

≥ 31.5 13.3 0.377 0.237 < 0.01 9.9 0.330 0.167 < 0.01

≥ 32 7.8 0.262 0.040 < 0.05 ns ns ns ns

H&C + SC ≥ 30.5 9.2 0.261 0.646 < 0.01 6.2 0.206 0.545 < 0.05

≥ 31 12.8 0.330 0.418 < 0.01 6.6 0.215 0.315 < 0.05

≥ 31.5 11.6 0.308 0.206 < 0.01 9.4 0.282 0.140 < 0.01

≥ 32 7.4 0.222 0.033 < 0.05 ns ns ns ns

H&C + SC + UKI ≥ 30.5 13.7 0.313 0.639 < 0.001 11.3 0.287 0.582 < 0.01

≥ 31 23.2 0.436 0.442 < 0.001 15.4 0.355 0.381 < 0.001

≥ 31.5 19.1 0.389 0.229 < 0.001 15.5 0.357 0.186 < 0.001

≥ 32 13.9 0.317 0.060 < 0.001 11.0 0.283 0.056 < 0.01

MLRF ≥ 30 ns ns ns ns ns ns ns ns

≥ 30.5 ns ns ns ns 10.6 0.346 0.481 < 0.01

≥ 31 ns ns ns ns 4.5 0.176 0.050 < 0.05
